# Combining *R* gene and quantitative resistance increases effectiveness of cultivar resistance against *Leptosphaeria maculans* in *Brassica napus* in different environments

**DOI:** 10.1371/journal.pone.0197752

**Published:** 2018-05-23

**Authors:** Yong-Ju Huang, Georgia K. Mitrousia, Siti Nordahliawate M. Sidique, Aiming Qi, Bruce D. L. Fitt

**Affiliations:** Centre for Agriculture, Food & Environmental Management, School of Life and Medical Sciences, University of Hertfordshire, Hatfield, Hertfordshire, United Kingdom; Universita degli Studi di Pisa, ITALY

## Abstract

Using cultivar resistance against pathogens is one of the most economical and environmentally friendly methods for control of crop diseases. However, cultivar resistance can be easily rendered ineffective due to changes in pathogen populations or environments. To test the hypothesis that combining *R* gene-mediated resistance and quantitative resistance (QR) in one cultivar can provide more effective resistance than use of either type of resistance on its own, effectiveness of resistance in eight oilseed rape (*Brassica napus*) cultivars with different *R* genes and/or QR against *Leptosphaeria maculans* (phoma stem canker) was investigated in 13 different environments/sites over three growing seasons (2010/2011, 2011/2012 and 2012/2013). Cultivar Drakkar with no *R* genes and no QR was used as susceptible control and for sampling *L*. *maculans* populations. Isolates of *L*. *maculans* were obtained from the 13 sites in 2010/2011 to assess frequencies of avirulent alleles of different effector genes (*AvrLm1*, *AvrLm4* or *AvrLm7*) corresponding to the resistance genes (*Rlm1*, *Rlm4* or *Rlm7*) used in the field experiments. Results of field experiments showed that cultivars DK Cabernet (*Rlm1* + QR) and Adriana (*Rlm4* + QR) had significantly less severe phoma stem canker than cultivars Capitol (*Rlm1*) and Bilbao (*Rlm4*), respectively. Results of controlled environment experiments confirmed the presence of *Rlm* genes and/or QR in these four cultivars. Analysis of *L*. *maculans* populations from different sites showed that the mean frequencies of *AvrLm1* (10%) and *AvrLm4* (41%) were less than that of *AvrLm7* (100%), suggesting that *Rlm1* and *Rlm4* gene-mediated resistances were partially rendered ineffective while *Rlm7* resistance was still effective. Cultivar Excel (*Rlm7* + QR) had less severe canker than cultivar Roxet (*Rlm7*), but the difference between them was not significant due to influence of the effective resistance gene *Rlm7*. For the two cultivars with only QR, Es-Astrid (QR) had less severe stem canker than NK Grandia (QR). Analysis of the relationship between severity of stem canker and weather data among the 13 sites in the three growing seasons showed that increased severity of stem canker was associated with increased rainfall during the phoma leaf spot development stage and increased temperature during the stem canker development stage. Further analysis of cultivar response to environmental factors showed that cultivars with both an *Rlm* gene and QR (e.g. DK Cabernet, Adriana and Excel) were less sensitive to a change in environment than cultivars with only *Rlm* genes (e.g. Capitol, Bilbao) or only QR (e.g. DK Grandia). These results suggest that combining *R* gene and QR can provide effective, stable control of phoma stem canker in different environments.

## Introduction

Crop resistance against fungal pathogens has a vital role to play in ensuring global food security in response to the increasing human population and the threat from devastating diseases of food crops [1,2]. Crop resistance can be used to protect food crops grown by farmers who cannot afford the use of chemical fungicides in regions where crop production is being threatened by crop diseases and climate change [3]. Even in regions where farmers can afford the use of fungicides, fungicides can easily lose their effectiveness due to the development of fungicide insensitivity in pathogen populations [1,4–6]. Therefore, the need for effective crop resistance is greater than ever.

A problem with crop resistance against fungal pathogens is that it can be rendered ineffective through changes in pathogen populations [7–10]. Such changes can lead to ‘boom and bust’ cycles of crop production, when good years are followed by years with severe epidemics caused by new pathogen races. To ensure sustainable crop production, there is a need for durable crop resistance [11], defined as ‘a resistance that remains effective during its prolonged and widespread use in an environment favourable to the disease’. Effective resistance is resistance that prevents development of severe disease epidemics. Two types of resistance used to control crop diseases are quantitative resistance (QR) and *R* gene-mediated qualitative resistance. QR is usually controlled by several minor genes (quantitative trait loci; QTL), whereas qualitative resistance is usually controlled by single dominant *R* genes [12,13]. *R* gene-mediated resistance is race-specific; it is effective only when the avirulent allele of the corresponding effector gene is predominant in the pathogen population. Therefore, *R* gene-mediated resistance is not effective in protecting the crop when new pathogen races have developed, resulting in catastrophic epidemics [8,9,14]. Its effectiveness may also be influenced by environmental factors [15], such as temperature [16]. QR is considered race non-specific and more durable [13,17]. However, in the presence of large amounts of inoculum of different pathogen races in an environment favourable for disease development, QR cannot provide effective protection.

It has frequently been hypothesised that combining *R* gene-mediated resistance and quantitative resistance in one crop cultivar will provide more effective resistance across a range of environments than use of either type of resistance on its own. Evidence to support this hypothesis has been provided by Brun et al. [17] using the *Brassica napus* (oilseed rape; canola)-*Leptosphaeria maculans* (phoma stem canker; blackleg) host-pathogen system; this disease is responsible for global yield losses estimated at more than £1000M per growing season [18]; both quantitative and *R* gene-mediated resistance operate against *L*. *maculans* [13]. Huang et al. [19] showed that resistance mediated by QR can be environmentally stable. However, this work was done using the Darmor-*bzh* x Yudal mapping population, which does not segregate for *R* genes that are effective in the UK or France. Since there are different types of *R* genes [20] and different types of quantitative resistance [21], there is a need to test this hypothesis with material carrying different *R* genes. Previous work done by Brun et al. [17] was only on one *R* gene (*Rlm6*) which has never been used in commercial cultivars, and was only at one site/environment in several growing seasons. Therefore, there is a need to test this hypothesis with different *R* genes used in commercial cultivars in different environments over several growing seasons. This paper describes work with *B*. *napus-L*. *maculans* using three different *R* genes (*Rlm* genes) against *L*. *maculans* and different sources of quantitative resistance to investigate the impact of combining QR with *Rlm* gene-mediated resistance on effectiveness of oilseed rape cultivar resistance in different environments.

## Materials and methods

### Effectiveness of different cultivar resistances in control of phoma stem canker epidemics in winter oilseed rape field experiments

To investigate effects of combining QR with *Rlm* gene-mediated resistance on effectiveness of control of phoma stem canker epidemics in different environments, nine oilseed rape cultivars were used in field experiments. Six cultivars (cvs) had an *Rlm* gene in a background with or without QR: Capitol (*Rlm1*), DK Cabernet (*Rlm1* + QR), Bilbao (*Rlm4*), Adriana (*Rlm4* + QR), Roxet (*Rlm7*), Excel (*Rlm7* + QR). Two cultivars had QR without known *Rlm* genes: Es-Astrid and NK Grandia. Cultivar Drakkar (with no known *Rlm* genes and no QR) was used as a susceptible control and for sampling *L*. *maculans* populations. Field experiments were done at 13 sites (11 sites in the UK and one site each in France and Germany) in the 2010/2011, 2011/2012 and 2012/2013 growing seasons (Table 1).

**Table 1:** The latitude and longitude for field experiment sites and the associated weather stations over three growing seasons (2010/11, 2011/12 and 2012/13).

Field experiment			Weather			Harvest years with disease data
Site	Latitude (°)	Longitude (°)	Station	Latitude (°)	Longitude (°)	
Bainton, UK	53.97N	0.55W	High Mowthorpe	54.10N	0.64W	2011, 2013
Banbury, UK	52.11N	1.29W	Wellesbourne	52.20N	1.60W	2011, 2012, 2013
Cowlinge, UK	52.16N	0.46E	Broom's Barn	52.26N	0.56E	2011, 2012, 2013
Harpenden, UK	51.81N	0.36W	Rothamsted	51.80N	0.35W	2011, 2012, 2013
Harper Adams, UK	52.78N	2.43W	Newport	52.77N	2.42W	2012
Horncastle, UK	53.05N	0.54W	Cranwell	53.03N	0.50W	2012, 2013
Morley, UK	52.56N	1.04E	Norwich Airport	52.67N	1.28E	2011, 2012, 2013
Oadby Lodge, UK	52.60N	1.05W	Sutton Bonington	52.83N	1.25W	2012
Rothwell, UK	53.39N	0.57W	Waddington	53.18N	0.52W	2011, 2012, 2013
Spalding, UK	52.75N	0.19W	Wittering	52.37N	0.27W	2011, 2012, 2013
Stockbridge, UK	51.10N	1.57W	Middle Wallop	51.14N	1.56W	2011, 2013
Bad-Salzuflen, Germany	52.08N	8.70E	Bad-Salzuflen	52.08N	8.70E	2011, 2012, 2013
Verpillieres, France	49.67N	2.83E	Verpillieres	49.67N	2.83E	2011, 2012, 2013

In each growing season, seeds of the nine oilseed rape cultivars were sown in late August or early September, with a plot size of 10 m × 2 m at 12 sites and 15 m × 2 m at one site (Harpenden). No specific permissions were required for these locations of the field experiments because the field experiments were run by breeding companies on their own land or on subcontracted farms. The field experiments did not involve endangered or protected species because all the nine cultivars used were commercial oilseed rape cultivars. The field experiments were arranged in randomised block designs with three replicates. The severity of phoma stem canker was assessed in summer (late June to early July) before harvest by pulling up 20–30 plants (number of plants assessed varied between sites) from each plot, cutting the stem base of each plant and scoring the necrotic tissue in the cross-section using a 0−6 scale: 0, no affected tissue; 1, 1− 5% area affected, 2, 6 − 25% area affected; 3, 26−50% area affected; 4, 51–75% area affected; 5, 76–100% area affected, plant alive; 6, 100% area affected, stem broken or plant dead; modified from the 1–6 scale of Lô-Pelzer et al. [22]. At the end of the experiments, all the plots were harvested for yield.

### Temperature and rainfall at different field experiment sites

Daily rainfall, air maximum and minimum temperature data were obtained from the closest weather station for each experimental site in the UK or from on-site weather stations in France and Germany (Table 1). Mean daily air temperature was calculated as mean of minimum and maximum temperatures. Then the monthly mean air temperature and monthly total rainfall were calculated. The mean air temperature and total rainfall were also calculated for combined months of August to September (Aug-Sept), October to November (Oct-Nov), December to March (Dec-Mar) and April to June (Apr-Jun). Aug-Sept represented environmental conditions in summer for pseudothecial maturation (i.e. the development of sexual ascospores in pseudothecia) [23], whereas Oct-Nov & Dec-Mar represented environmental conditions in autumn/winter for the phoma leaf spot development stage, and Apr-Jun represented environmental conditions in spring/summer for the phoma stem canker development stage [19,24].

### Detection of avirulent alleles of different effector genes in *L*. *maculans* populations at different field experiment sites

To investigate the effectiveness of different *Rlm* genes (i.e. *Rlm1*, *Rlm4* and *Rlm7*) in different cultivars used in field experiments at different sites, it is necessary to know the proportions of the avirulent alleles of the corresponding effector genes (i.e. *AvrLm1*, *AvrLm4* and *AvrLm7*) in *L*. *maculans* populations at each field experiment site. To sample the *L*. *maculans* populations, cultivar Drakkar was used as a ‘trap’ cultivar for *L*. *maculans* (since Drakkar has no known *Rlm* genes, it can be colonised by all the races of *L*. *maculans* present in the region) [25]. In the first growing season (2010/2011), leaves of Drakkar with phoma leaf spots were sampled in autumn from each field site. Leaves were washed with running tap water and blotted dry. A small piece of phoma leaf spot lesion was cut from each leaf and placed in a Petri dish lined with moist filter paper. The lid of the Petri dish was sprayed with distilled water to maintain high humidity. Petri dishes with leaf lesions were then incubated at 20°C with alternating 12h light/12 darkness. Mature pycnidia with pink cirrhi (asexual conidial spore masses) were produced on leaf lesions after 2–3 days of incubation. Single pycnidial isolates of *L*. *maculans* were obtained by plucking off the cirrhus from a single pycnidium using a fine needle and transferring it to a drop of sterilized water to make a spore suspension. The conidial spore suspension was then pipetted onto a plate containing V8 medium amended with 20 units ml^-1^ penicillin and 40 units ml^-1^ streptomycin. After 3–5 days of incubation at 20°C in darkness, colonies of *L*. *maculans* developed; each colony represented an isolate from a single pycnidium from one leaf lesion. The isolates obtained were sub-cultured for making conidial suspensions. The concentrations of conidial suspensions were adjusted to 10^7^ spores mL^-1^ and they were stored at –20°C ready for inoculation.

Avirulent alleles of effector genes in each *L*. *maculans* isolate were identified by inoculating the isolate onto cotyledons of a differential set of cultivars/lines carrying known *Rlm* genes [26]. The differential set consisted of cultivars/lines Westar (no known *Rlm* gene against *L*. *maculans*, susceptible control), Columbus (*Rlm1*, *Rlm3*), Bristol (*Rlm2*, *Rlm9*), Jet Neuf (*Rlm4*), 150-2-1 (*Rlm5*), Darmor-MX (*Rlm6*), 23-1-1 (*Rlm7*) and 190-1-1 (*Rlm9*). Apart from Darmor-MX (provided by Dr Regine Deourme, INRA, Le Rheu, France), all the other cultivars/lines were provided by Dr Marie-Helene Balesdent (INRA-Bioger, Thiverval-Grignon, France). Cotyledons of two-week old seedlings of the differential set were wounded using a sterile needle and a 10μL drop of conidial suspension was placed over each wound. Each isolate was inoculated onto five to eight plants of each cultivar/line. Trays with inoculated plants were covered with a plastic propagator lid for 72 h to maintain high humidity and incubated at 20°C with alternating 12h light (light intensity 210μmol m^-2^s^-1^) and 12h darkness. At 17–20 days post inoculation (dpi), disease symptoms on each plant were scored on a 0–9 scale (0, no darkening around the wound; 9, large pale grey lesion with pycnidia) [27]. Isolates producing a mean score < 3.5 were considered avirulent (i.e. the isolate is avirulent in the presence of the corresponding *Rlm* gene), while isolates producing a score ≥3.5 were considered virulent.

### Detection of *Rlm* genes and quantitative resistance in different cultivars in controlled-environment experiments

To confirm the presence of resistance genes *Rlm1*, *Rlm4* or *Rlm7* in cultivars Capitol, DK Cabernet, Bilbao, Adriana, Roxet and Excel, cotyledons of two-week old seedlings of these cultivars were wounded and inoculated with conidia of *L*. *maculans* isolates carrying the avirulent (*Avr*) alleles of the corresponding effector genes (i.e. *AvrLm1*, *AvrLm4* or *AvrLm7*) (Table 2) using the method described above. At 18 dpi, disease symptoms were scored on the 0–9 scale; a mean score < 3.5 indicated the presence of the corresponding *Rlm* gene.

**Table 2:** Oilseed rape (*Brassica napus*) cultivars/lines and *Leptosphaeria maculans* isolates used in the controlled environment experiments to confirm the presence of *Rlm* genes or quantitative resistant (QR).

**Isolate** **Cultivar/line^a^**	**99–79^b^** **(**Av2**-4-7)**	**V23.2.1** **(**Av**4**-5-6**-7)**	**V23.11.9** **(**Av**1**-5-6**-7)**	**ME24** **(**Av**1**-6-7**)**
Adriana (*Rlm4* +QR)	CI^c^	CI	CI, PI^d^	NT^e^
Bilbao (*Rlm4*)	CI	CI	CI, PI	NT
Capitol (*Rlm1*)	NT	CI, PI	CI	CI
DK Cabernet (*Rlm1* +QR)	NT	CI, PI	CI	CI
Excel (*Rlm7* +QR)	CI	CI	CI	CI
Roxet (*Rlm7*)	CI	CI	CI	CI
A30 (no *R*, no QR)	CI	NT	CI, PI	CI
C119 (QR)	CI	NT	CI, PI	CI

^a^ A30 and C119 are doubled haploid lines from the Darmor-*bzh* × Yudal mapping population (Pilet et al., 1998; Huang et al., 2014); all the others are commercial cultivars.

^b^ *L*. *maculans* isolates carrying different avirulent (*Avr*) alleles of *AvrLm* effector genes for detecting the corresponding host resistance (*R*) genes in cultivars. The *Avr* alleles in bold numerals indicate isolates for detecting corresponding *Rlm* genes (e.g. *Rlm1*, *Rlm4* or *Rlm7*).

^c^ Cotyledon inoculation (CI) with the *L*. *maculans* isolate carrying the avirulent allele of the *AvrLm* effector gene to confirm the presence of corresponding *Rlm* gene.

^d^ Petiole inoculation (PI) with *L*. *maculans* isolate carrying the virulent allele of the *AvrLm* effector gene (i.e. to avoid the effect of *Rlm* gene resistance) to assess the effectiveness of quantitative resistance.

^e^ Not tested.

To estimate the differences between Capitol (*Rlm1*) and DK Cabernet (*Rlm1* + QR) or between Bilbao (*Rlm4*) and Adriana (*Rlm4* + QR) in terms of background quantitative resistance (QR), leaf petioles of the four cultivars were inoculated to assess stem canker severity using the methods described by Huang et al. [28]. To assess quantitative resistance in these four cultivars, it was necessary to use isolates virulent against *Rlm1* or *Rlm4* so that the effects of *Rlm* gene resistance could be avoided. Isolate v23.11.9 (Av1-5-6-7; avirulent against *Rlm1*, virulent against *Rlm4*) was used to inoculate leaf petioles of Bilbao and Adriana; isolate v23.2.1 (Av4-5-6-7; avirulent against *Rlm4*, virulent against *Rlm1*) was used to inoculate leaf petioles of Capitol and DK Cabernet (Table 2). Doubled haploid (DH) line A30 was used as a susceptible control and line C119 (with good quantitative resistance) [28,29] was used as a resistant control. Plants were grown in pots (9 cm diameter) with one plant per pot. The petioles of the first three leaves of each plant were inoculated when the plants had three fully expanded leaves. The experiment was arranged as a randomised block design with six blocks. The lower part of the leaf petiole was wounded using a sterile pin and a 10 μL droplet of conidial suspension was placed over the wound. After inoculation, plants in trays were covered with tray lids to maintain high relative humidity for 72 h. Inoculated plants were kept at 20°C with alternating 12h light/12 h darkness. At 49 dpi, stems of the inoculated plants were sampled by cutting each stem at the soil surface. The stems were then cut horizontally at the leaf scar of the inoculated leaf to assess the internal severity of stem canker on the 0–6 scale (see above). Then the stem was cut vertically and the length of internal symptoms along the stem cortex/pith was measured.

### Statistical analysis

For field experiments at 13 sites over the three growing seasons, analysis of variance was done with the data for severity of stem canker to assess the differences between cultivars, between sites and between seasons. For each growing season, the cultivar mean phoma stem canker severity score was calculated from the three replicates and the site mean phoma stem canker severity score was calculated from the nine cultivars at each site. To assess cultivar response to environmental factors (e.g. pathogen inoculum abundance, pathogen race structure composition, rainfall and temperature) within experimental sites, the relative cultivar stem canker severity (RS), expressed as a percentage, was calculated by dividing a given cultivar stem canker severity score by the mean stem canker severity score at a given site (i.e. the mean score of all cultivars at that site) and analysis of variance was also done to assess differences between different cultivars in relative stem canker severity.

To assess cultivar response to environmental factors at different experimental sites, the relationships between cultivar stem canker severity score and site mean stem canker severity score were analysed using linear regression. Similar analyses were done with data for yield of different cultivars. For controlled environment experiments, data for stem canker severity score and lengths of internal symptoms were analysed by analysis of variance (ANOVA) to assess the differences between cultivars. All the analyses were done using GENSTAT statistical software [30].

## Results

### Effectiveness of different cultivar resistances in control of phoma stem canker epidemics in field experiments

**Fig 1:**
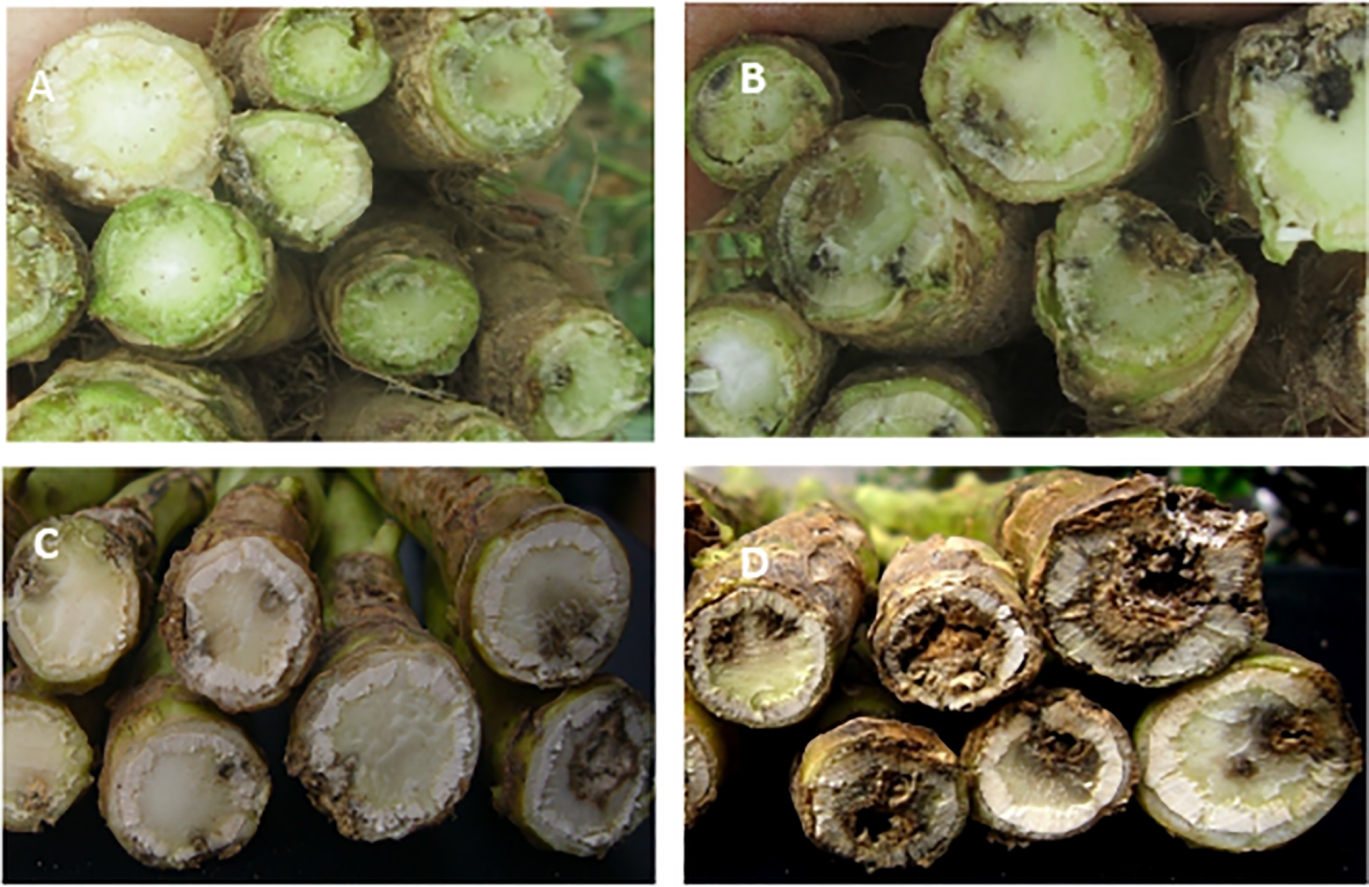
Phoma stem canker symptoms on plants from a winter oilseed rape field experiment. Stems of cultivar Adriana (A) (*Rlm4*+QR) with resistance gene *Rlm4* and quantitative resistance (QR) had less severe stem canker than cultivar Bilbao (B) (only *Rlm4*); similarly those of cultivar DK Cabernet (C) (*Rlm1*+QR) had less severe stem canker than cultivar Capitol (D) (*Rlm1*). Stems were sampled from the field experiment at Morley, Norfolk on 8 July 2013.

The analysis of variance showed that there were significant differences in phoma stem canker severity between cultivars (*P*<0.01, 8 d.f., SED 0.27). The phoma stem canker severity score was the smallest on Excel (0.85) (*Rlm7* + QR), but did not differ significantly from that on Roxet (0.95) (*Rlm7*) (Table 3). DK Cabernet (1.39) (*Rlm1* + QR) had significantly less severe phoma stem canker than Capitol (2.42) (*Rlm1*). Similarly, Adriana (1.42) (*Rlm4* + QR) had significantly less severe phoma stem canker than Bilbao (2.13) (*Rlm4*) (Table 3). However, phoma stem canker severity did not differ significantly between Capitol and Bilbao or between DK Cabernet and Adriana. It was often observed that stem canker symptoms had spread into the pith of Capitol and Bilbao, while symptoms were mainly restricted to the cortex or had just started to spread into the pith of Adriana and DK Cabernet before harvest (Fig 1). For the two cultivars with QR only, Es-Astrid (1.09) had significantly less severe stem canker than NK Grandia (1.73) (Table 3).

**Table 3:** Comparison of phoma stem canker severity and seed yield between different oilseed rape cultivars grown in winter oilseed rape field experiments at 13 sites over three growing seasons (2010/2011, 2011/2012, 2012/2013).

Cultivar^a^	Phoma stem canker		Seed yield	
	Severity^b^	Relative severity (%)^c^	Yield (t/ha)	Relative yield (%)^c^
Adriana (*Rlm4* + QR)	1.42de^d^	68.5de	4.28bc	106.20c
Bilbao (*Rlm4*)	2.13bc	118bc	4.40ab	109.19bc
Capitol (*Rlm1*)	2.42b	136.7b	3.52d	87.30e
DK Cabernet (*Rlm1* + QR)	1.39de	69.6de	4.56ab	114.17ab
Drakkar (no *R*, no QR)	4.26a	277.4a	1.96e	48.20f
Es-Astrid (QR)	1.09ef	51.4ef	4.49ab	111.96bc
Excel (*Rlm7* + QR)	0.85f	36.2f	4.79a	118.87a
NK Grandia (QR)	1.73cd	96.3c	4.39ab	109.07bc
Roxet (*Rlm7*)	0.95ef	45.8ef	3.84cd	95.04d
SED^e^	0.27	12.95	0.22	3.28

^a^ Oilseed rape cultivars with or without an *Rlm* gene against *Leptosphaeria maculans* in a background with or without quantitative resistance (QR) were used in field experiments.

^b^The severity of phoma stem canker was assessed in summer before harvest on a 0−6 scale modified from that of Lô-Pelzer et al. (2009).

^c^The relative cultivar stem canker severity or seed yield was calculated as a percentage, based on the relationships between stem canker severity score or seed yield for individual cultivars and the mean stem canker severity score or yield for all cultivars at a given site.

^d^Different letters indicate a significant difference at *P* ≤0.05.

^e^SED is the standard error of the difference used to calculate the least significance difference (LSD) at significance level *P* ≤0.05 when multiple comparisons were done.

There were significant differences in phoma stem canker severity between growing seasons (*P*<0.01, 2 df, SED 0.05) and sites (*P*<0.01, 12 d.f., SED 0.08). Phoma stem canker was more severe in 2011 (2.2) than in 2012 (1.5) or 2013 (1.7). Among the field sites in the UK, Morley (2.7) and Harpenden (2.7) had the greatest phoma stem canker severity scores, followed by Cowlinge (2.4) and Bainton (2.2). The phoma stem canker severity score at Banbury (1.0), Spalding (1.2), Rothwell (1.6), Stockbridge (1.5) and Horncastle (1.2) was < 2.0. The phoma stem canker severity scores at Verpillieres in France (1.7) and Bad Salzuflen in Germany (1.1) were also < 2.0.

**Fig 2:**
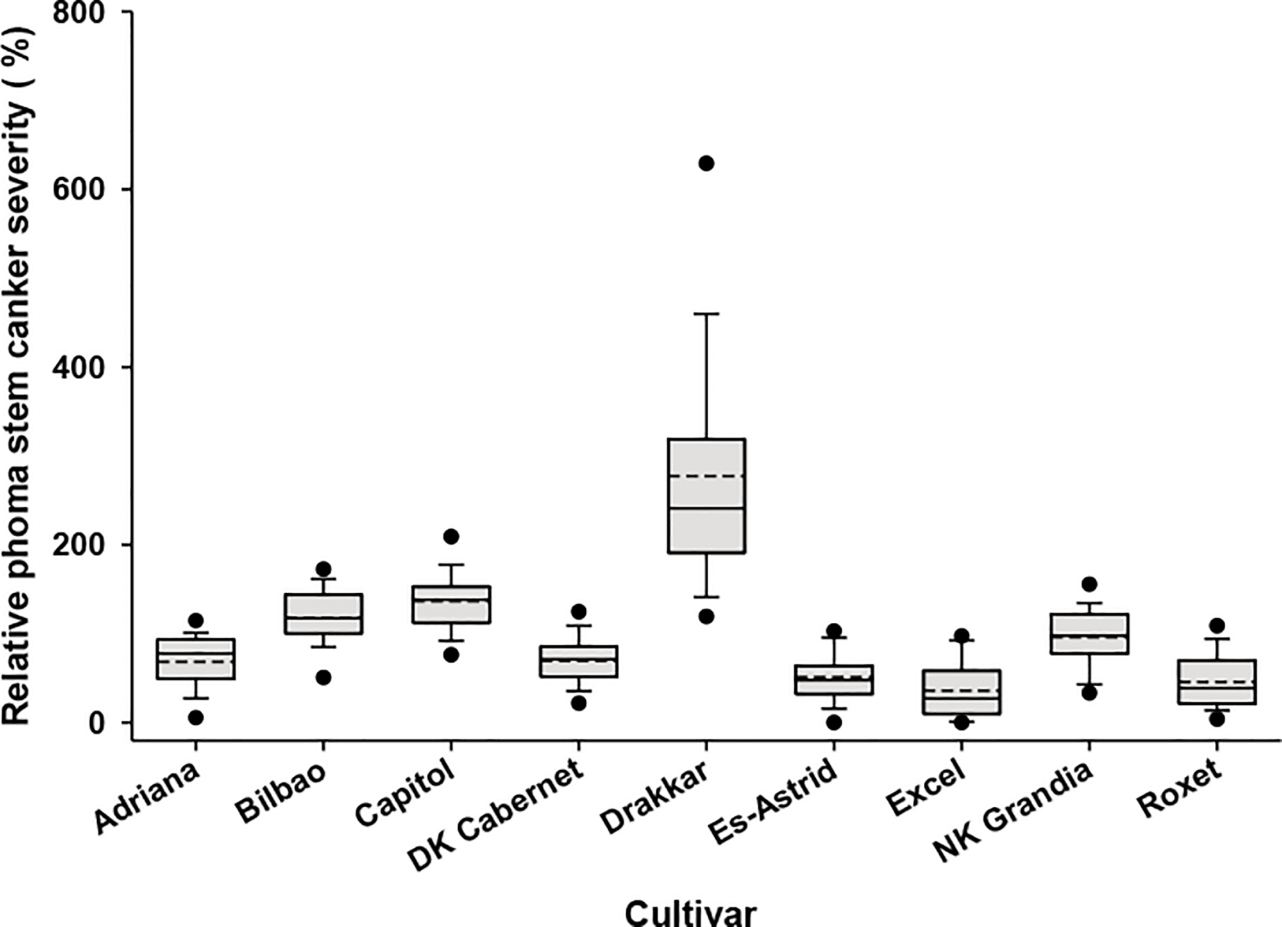
Distribution of relative phoma stem canker severity. The distribution of relative phoma stem canker severity for each cultivar from winter oilseed rape field experiments at 13 sites in three growing seasons (2010/11, 2011/12 and 2012/13). Each box-plot shows the mean (broken line) and median (solid line) percentage relative phoma stem canker severity. The lower and upper boundaries of the boxes indicate relative phoma stem canker severity for the 25^th^ and 75^th^ percentiles, while whisker bars below and above each box indicate relative phoma stem canker severity for the 5^th^ and 95^th^ percentiles. The black dots below and above each box-plot represent the minimum and maximum relative phoma stem canker severities, respectively.

Analysis of cultivar relative phoma stem canker severity (i.e. a measurement of cultivar response to environment across different experimental sites) showed that Drakkar (277.4%) had the greatest relative stem canker severity (Table 3). The relative stem canker severity was smallest on Excel (36.2%) and did not differ significantly from that on Roxet. The relative phoma stem canker severity was significantly smaller on DK Cabernet and Adriana than on Capitol and Bilbao (Table 3). The distribution of relative phoma stem canker severity showed that it was most variable on Drakkar, while it was least variable on Es-Astrid across different sites (Fig 2).

**Fig 3:**
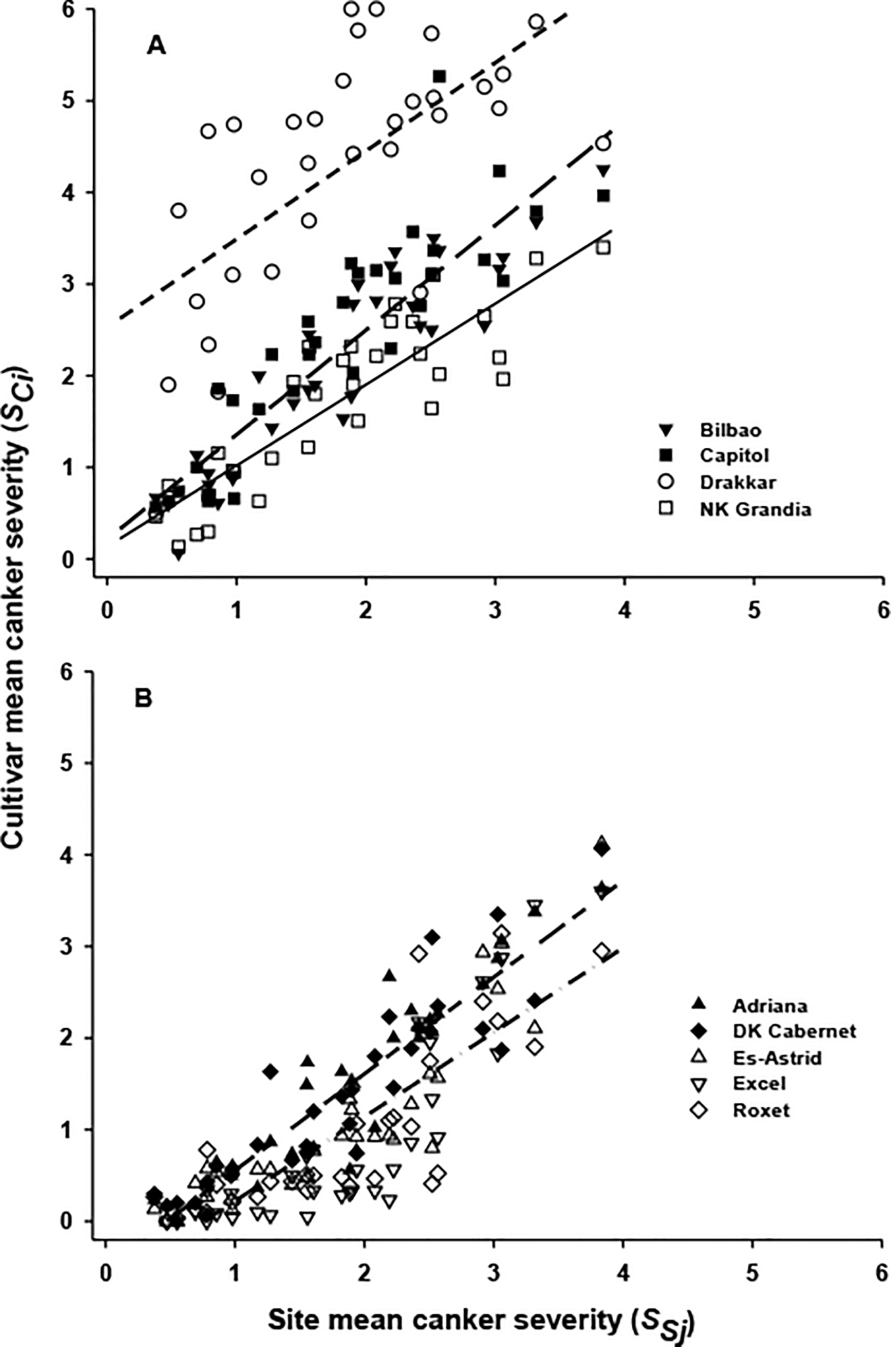
Relationships between cultivar phoma stem canker severity (*S*_*Ci*_) and site mean phoma stem canker severity (*S*_*Sj*_). The relationships between *S*_*Ci*_ and *S*_*Sj*_ in winter oilseed rape field experiments at 13 sites in three growing seasons (2010/11, 2011/12 and 2012/13). Analysis of position and parallelism showed five distinct groups, with three groups in Fig 3A: Drakkar (broken short line, *S*_*C*_ = 2.53+0.96*S*_*S*_), Capitol and Bilbao (broken long line, *S*_*C*_ = 0.22+1.14*S*_*S*_), NK Grandia (solid line, *S*_*C*_ = 0.14+0.88*S*_*S*_) and with two groups in Fig 3B: Adriana and DK Cabernet (broken long-short line, *S*_*C*_ = -0.50+1.05*S*_*S*_), Ex-Astrid, Roxet and Excel (dash-dot line, *S*_*C*_ = -0.70+0.92*S*_*S*_).

Analysis of the relationship between the cultivar phoma stem canker severity (*S*_*Ci*_) and the site mean phoma stem canker severity (*S*_*Sj*_) for each cultivar showed a linear relationship. When an analysis of position and parallelism was done for all cultivars, five distinct groups were identified (Fig 3) and the percentage variance in the cultivar phoma stem canker severity accounted for by the site mean phoma stem canker severity was 84.3% (282 d.f.). Drakkar alone formed a group (*S*_*C*_ = 2.53+0.96*S*_*S*_; Fig 3A), which was the most sensitive to a change in environment, followed by the group consisting of Capitol and Bilbao (*S*_*C*_ = 0.22+1.14*S*_*S*_). NK Grandia alone formed a group (*S*_*C*_ = 0.14+0.88*S*_*S*_). Adriana and DK Cabernet formed one group (*S*_*C*_ = –0.50+1.05*S*_*S*_; Fig 3B) while Ex-Astrid, Roxet and Excel formed another group (*S*_*C*_ = –0.70+0.92*S*_*S*_); these two groups were the least sensitive to a change in environment.

### Temperature and rainfall in relation to phoma stem canker severity in field experiments

Monthly mean temperature and monthly total rainfall differed between growing seasons at a given site and differed between sites within a given season. Based on the calculated standard deviation and coefficient of variation, the monthly mean temperature varied most in the winter months, followed by autumn months, but varied least in summer months (S1 Table). However, the coefficient of variation in the monthly total rainfall was within a narrow range at *c*. 50% between months during the growing season, except in March & April (S2 Table). A simple correlation coefficient was calculated for each cultivar to examine the relationship between the phoma stem canker score and each of the mean temperature and total rainfall in Aug-Sept, Oct-Nov, Dec-Mar and Apr-Jun (Table 4; S3 Table). It is clear that the phoma stem canker severity was related to both rainfall and temperature for all cultivars. In general, increased phoma stem canker severity was associated with increased rainfall in Aug-Sept, Oct-Nov and Dec-Mar (ascospore maturation and phoma leaf spot development stage) and with increased temperature in Apr-Jun (phoma stem canker development stage).

**Table 4:** The simple correlation coefficients calculated between stem canker score at harvest and the mean temperature (°C) or the total rainfall (mm) over three growing seasons (2010/2011, 2011/2012, 2012/2013) at 13 sites.

Variable	Months	Adriana	Bilbao	Capitol	DK Cabernet	Drakkar	Es-Astrid	Excel	NK Grandia	Roxet
**Temperature**	Aug-Sept	-0.14	-0.12	-0.25	-0.21	-0.03	-0.10	-0.13	-0.02	0.02
	Oct-Nov	-0.27	-0.17	-0.26	-0.24	0.03	-0.13	-0.09	-0.08	-0.10
	Dec-Mar	-0.16	-0.10	-0.14	-0.06	0.09	-0.04	0.01	0.05	-0.02
	Apr-Jun	0.19	0.13	0.09	0.16	-0.12	0.11	0.05	0.26	0.08
**Rainfall**	Aug-Sept	0.35	0.34	0.42	0.35	0.09	0.19	0.15	0.33	0.07
	Oct-Nov	0.35	0.25	0.33	0.29	0.21	0.29	0.30	0.10	0.27
	Dec-Mar	0.31	0.30	0.21	0.12	0.28	0.17	0.20	0.11	0.29
	Apr-Jun	-0.38	-0.19	-0.29	-0.36	-0.03	-0.27	-0.20	-0.19	-0.23

### Cultivar seed yield in relation to phoma stem canker severity in field experiments

**Fig 4:**
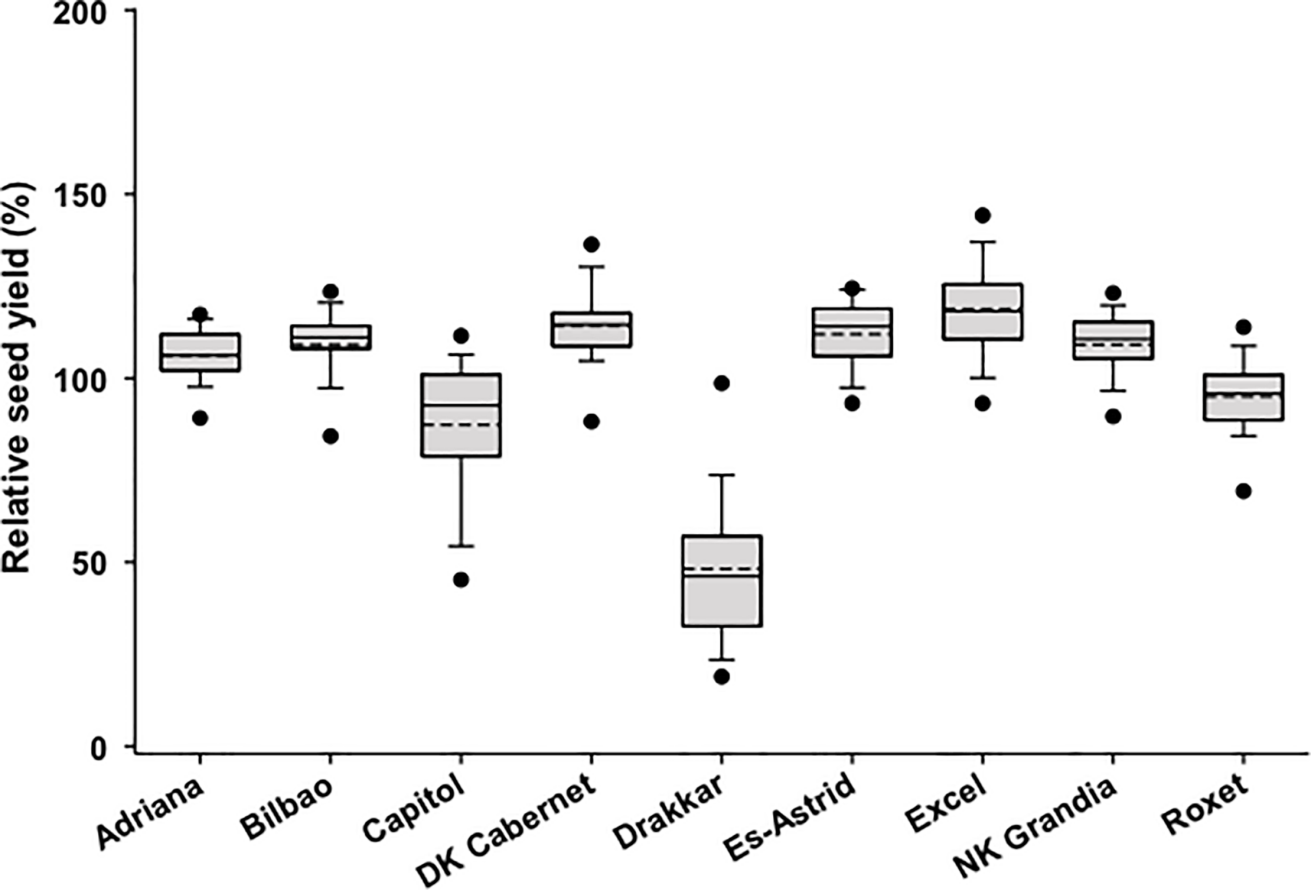
Distribution of relative seed yield. The distribution of relative seed yield for each cultivar from field experiments at 13 sites in three growing seasons (2010/11, 2011/12 and 2012/13). Each box-plot shows the mean (broken line) and median (solid line) relative seed yield. The lower and upper boundaries of the boxes indicate relative seed yield for the 25^th^ and 75^th^ percentiles, while whisker bars below and above each box indicate relative seed yield for the 5^th^ and 95^th^ percentiles. The black dots below and above each box-plot represent the minimum and maximum seed yields, respectively.

Analysis of variance showed that there were significant differences in seed yield between cultivars (*P*<0.01, 8 d.f., SED 0.22). Excel had the greatest yield (4.79 t/ha) and Capitol had smallest yield (3.52 t/ha) apart from Drakkar (Table 3). Excel had the greatest relative yield but it did not significantly differ from that of DK Cabernet. There were no significant differences between Adriana and Bilbao or Es-Astrid and NK Grandia in either yield or relative yield, but there were significant differences between DK Cabernet and Capitol (Table 3). Analysis of the distribution of relative seed yield (i.e. the ratio of cultivar yield to site mean yield; an indicator of cultivar yield response to environmental factors) showed that yields of Capitol and Drakkar were the most variable while yield of Bilbao was the most stable (Fig 4).

**Fig 5:**
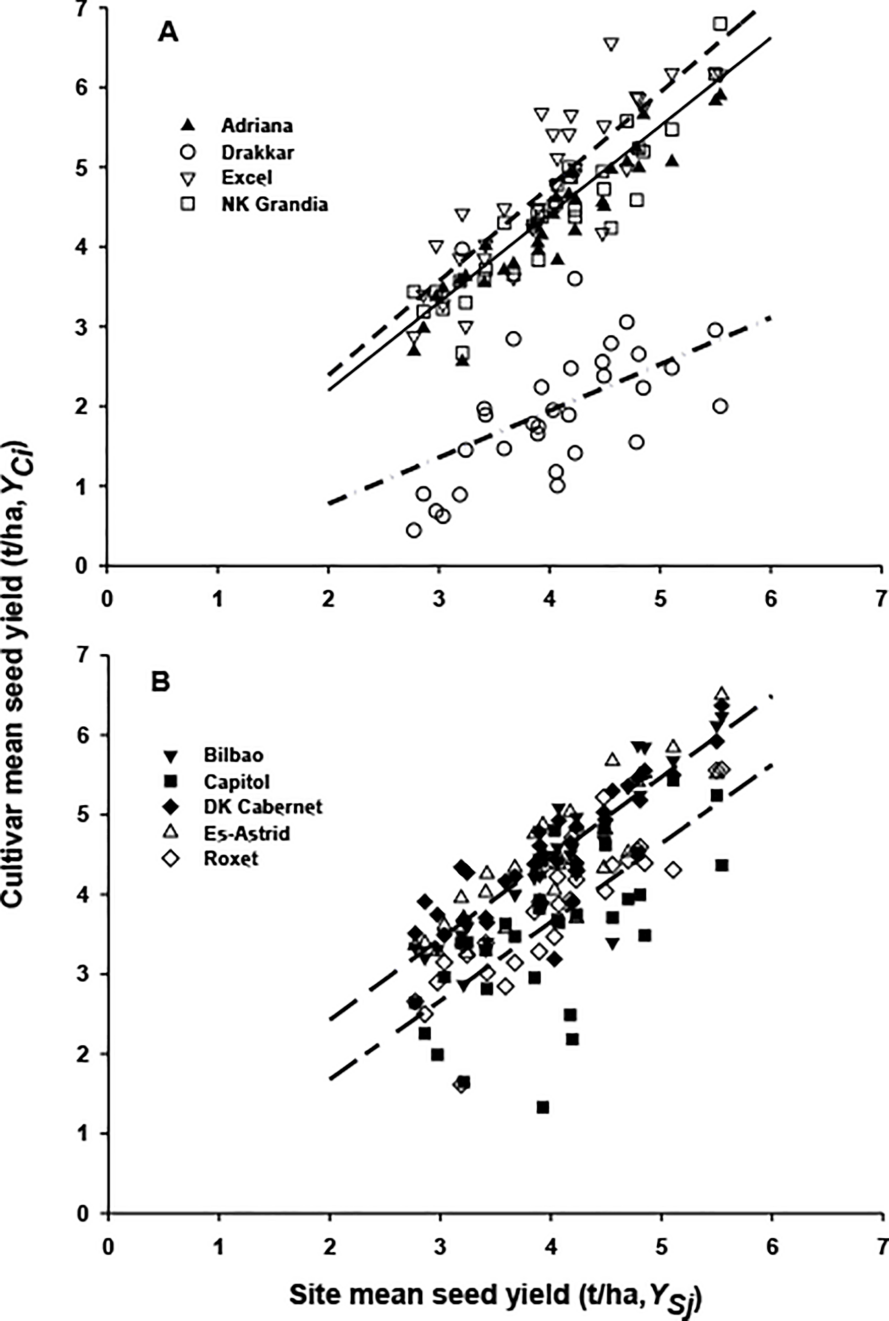
Relationship between individual cultivar seed yield (*Y*_*Ci*_) and site mean seed yield (*Y*_*Sj*_). The relationship between *Y*_*Ci*_ and *Y*_*Sj*_ in winter oilseed rape field experiments at 13 sites in three growing seasons (2010/11, 2011/12 and 2012/13). Analysis of position and parallelism showed five distinct groups, with three groups in Fig 5A: Excel (broken short line, *Y*_*C*_ = 0.03+1.18*Y*_*S*_), NK Grandia and Adriana (solid line, *Y*_*C*_ = -0.01+1.11*Y*_*S*_), Drakkar (dash-dot line, *Y*_*C*_ = -0.39 + 0.58*Y*_*S*_) and with two groups in Fig 5B: Ex-Astrid, DK Cabernet and Bilbao (broken long line, *Y*_*C*_ = 0.40+1.02*Y*_*S*_), Roxet and Capitol (broken long-short line, *Y*_*C*_ = -0.29+0.99*Y*_*S*_).

To study cultivar performance at different sites, the relationship between the cultivar seed yield (*Yc*) and site mean seed yield (*Ys*) was analysed for each cultivar. There was a simple linear relationship between the cultivar seed yield and site mean seed yield for each cultivar. When analysis of position and parallelism was done for all cultivars, five distinct groups were identified (Fig 5) and the percentage variance in cultivar seed yield accounted for by site mean seed yield was 82.4% (278 d.f.). The first group included only Excel (*Y*_*C*_ = 0.03+1.18*Y*_*S*_; Fig 5A) which was the most responsive to the high potential yield at a given site in a given growing season. The second most responsive group of cultivars included Es-Astrid, DK Cabernet and Bilbao (*Y*_*C*_ = 0.40+1.02*Y*_*S*_). These three cultivars yielded more than the site mean seed yield as the slope of the response line was greater than one. NK Grandia and Adriana were in the third group (*Y*_*C*_ = -0.01+1.11*Y*_*S*_) and they also yielded better than the site mean seed yield because the slope of the response line was greater than one. Roxet and Capitol were in the fourth group (*Y*_*C*_ = -0.29+0.99*Y*_*S*_; Fig 5B) and they yielded less than the site mean seed yield because the slope of the response line was smaller than one. The susceptible Drakkar was in the last group, which was most responsive to unfavourable environment (*Y*_*C*_ = -0.39+0.58*Y*_*S*_).

Analysis of the effect of phoma stem canker severity on yield showed that cultivar seed yield (*Y*_*C*_) was negatively correlated with the cultivar phoma stem canker severity (*S*_*C*_) (*Y*_*C*_ = 4.72–0.39*S*_*C*_). The correlation coefficient was statistically significant (*P*<0.01, *r* = –0.47, 286 d.f.). However, the simple linear relation accounted for only 21.9% of the variation in the cultivar mean seed yield by cultivar mean phoma stem canker severity. Therefore, the relationship was further analysed using relative cultivar yield and relative cultivar phoma stem canker severity.

**Fig 6:**
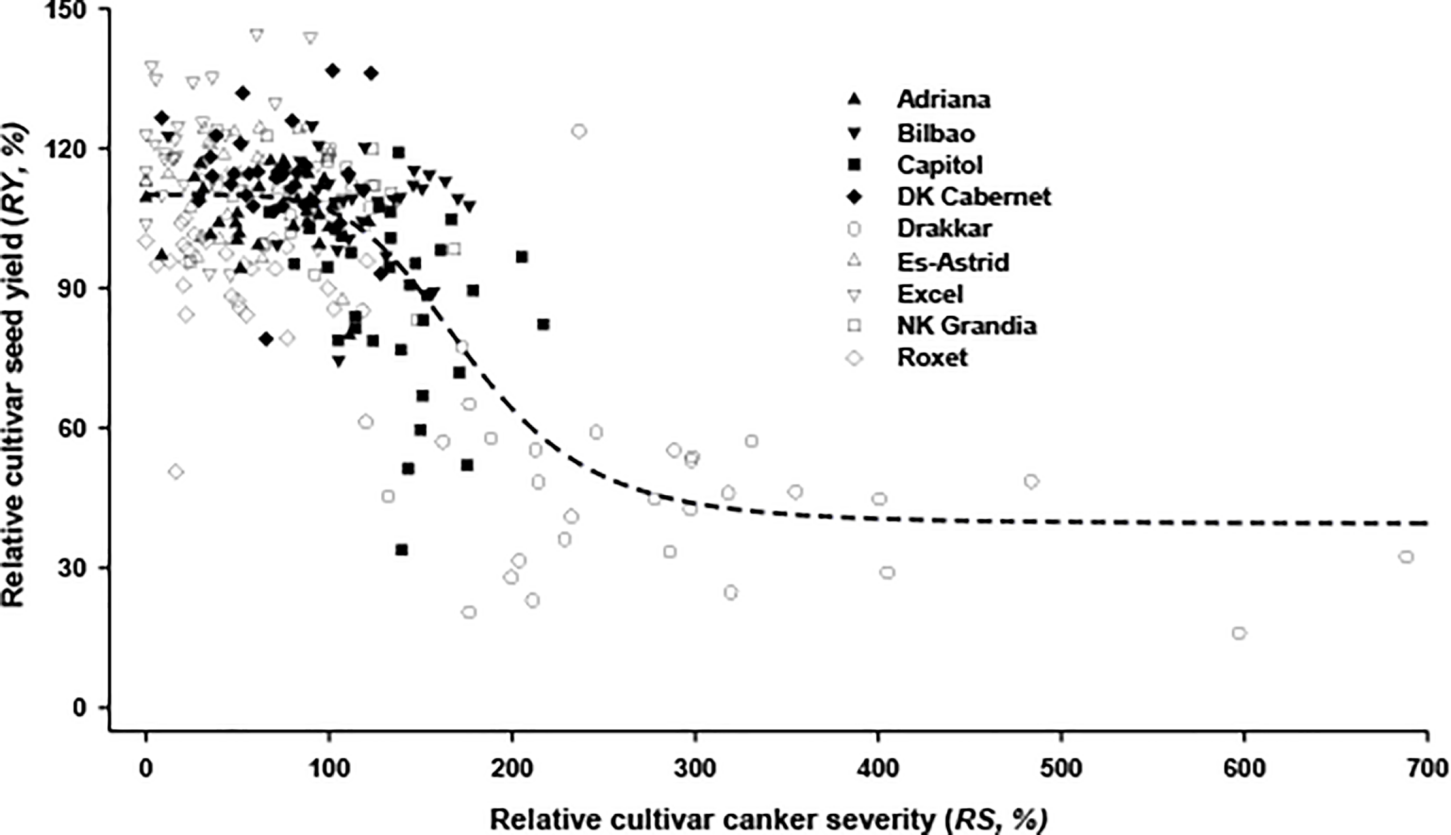
Relationship between relative cultivar seed yield (*RY*, %) and relative cultivar phoma stem canker severity (*RS*, %). The relationship was fitted (broken short line) by the logistic equation ($RY=39.54+\frac{70.50}{1+{0.0056(RS)}^{5.23}}$) using the data from winter oilseed rape field experiments at 13 sites (see Table 1) in three growing seasons (2010/2011, 2011/2012, 2012/2013).

The relative cultivar seed yield (*RY*) decreased with increasing relative cultivar phoma stem canker severity (*RS*) and then reached an asymptote when the relative cultivar phoma stem canker severity increased further (Fig 6). The correlation coefficient was statistically significant (*P*<0.01, *r* = –0.69, 286 d.f.). A logistic equation fitted best to the data to describe this relationship ($RY=39.54+\frac{70.50}{1+{0.0056(RS)}^{5.23}}$). The fitted logistic equation accounted for 47.2% of the variation in relative cultivar seed yield by relative cultivar phoma stem canker severity. Therefore, the remaining 53% of the variation in relative cultivar seed yield was accounted for by other factors affecting yield.

### Detection of avirulent alleles of different effector genes in *L*. *maculans* populations at different field experiment sites

Leaves of Drakkar with phoma leaf spots were sampled between October and December 2010; from those leaf samples a total of 131 single pycnidial *L*. *maculans* isolates was obtained, with the number of isolates obtained from each site varying from five to 17 (Table 5). All these isolates were characterised at eight *Avr* loci (i.e. from *AvrLm1* to *AvrLm9* except for *AvrLm8*, since host material carrying *Rlm8* was not available).

**Table 5:** Characteristics of the race structure of *Leptosphaeria maculans* populations at different experimental sites in the UK, France and Germany.

**Site^a^**	**No. isolates**	**No. avirulent alleles per isolate**	**% isolates avirulent at each of five *Avr* loci^b^**				
			***AvrLm1***	***AvrLm4***	*AvrLm5*	*AvrLm6*	***AvrLm7***
Bainton, UK	6	4.0	16.7	83.3	100	100	100
Banbury, UK	11	2.8	9.1	18.2	54.5	100	100
Cowlinge, UK	9	3.7	11.1	11.1	100	100	100
Harpenden, UK	11	3.6	36.4	27.3	100	100	100
Harper Adams, UK	15	3.7	26.7	46.7	93.3	100	100
Morley, UK	10	4.0	10.0	80.0	100	100	100
Oadby Lodge, UK	15	3.6	26.7	33.3	100	100	100
Rothwell, UK	12	2.9	0	33.3	66.7	100	100
Spalding, UK	17	3.6	23.5	52.9	94.1	100	100
Stockbridge, UK	12	3.3	0	25.0	100	100	100
Bad-Salzuflen, Germany	5	2.6	0	0	100	60	100
Verpillieres, France	8	2.9	0	0	100	88.9	100

^a^ For details of the sites, see Table 1.

^b^Each isolate was characterised at eight *AvrLm* loci; all the isolates tested were virulent at *AvrLm2*, *AvrLm3* and *AvrLm9* loci, so these loci are not presented in this table. Avirulent alleles in bold correspond to the *Rlm* resistance genes (i.e. *Rlm1*, *Rlm4*, *Rlm7*) in the cultivars used in winter oilseed rape field experiments.

For all the *L*. *maculans* isolates collected from the UK (total 118), the frequencies of the avirulent alleles of *AvrLm6* and *AvrLm7* were 100% at all sites sampled (Table 5). The other most frequent avirulent allele in the UK *L*. *maculans* populations was *AvrLm5* (90.9%); the virulent allele of *AvrLm5* was detected at only four out of 10 sites. All the isolates tested were virulent at *AvrLm2*, *AvrLm3* and *AvrLm9* loci. There were differences between sites in frequency of avirulent alleles of *AvrLm1* and *AvrLm4*, with the mean frequency of *AvrLm4* (41.1%) greater than that of *AvrLm1* (10%). Avirulent allele *AvrLm4* was detected at all the sites with a frequency ranging from 11 to 83%, while avirulent allele *AvrLm1* was detected at eight sites with a frequency ranging from 9 to 36% (Table 5). Only avirulent alleles of *AvrLm5*, *AvrLm6* and *AvrLm7* were detected in *L*. *maculans* isolates from France and Germany. The mean number of avirulent alleles in the UK *L*. *maculans* isolates (3.5) was greater than that in French (2.9) and German (2.6) isolates. Most of the UK *L*. *maculans* isolates had 3–4 avirulent alleles.

Of the eight *Avr* loci characterised for UK *L*. *maculans* isolates, only three of them (*AvrLm1*, *AvrLm4* and *AvrLm5*) varied between different sites. There were only seven races (Av5-7, Av6-7, Av4-6-7, Av5-6-7, Av1-5-6-7, Av4-5-6-7, Av1-4-5-6-7) identified in the population. The races Av5-6-7 and Av4-5-6-7 were detected at all sites sampled, and these two races represented 72% of the UK *L*. *maculans* population in 2010. The races Av1-5-6-7 and Av1-4-5-6-7 represented 8% and 10% of the UK *L*. *maculans* population, respectively. The other three races were present at a frequency <5% in the UK *L*. *maculans* population. Only limited numbers of isolates from France and Germany were characterised; there were only two races (Av5-7 and Av5-6-7) identified, with the frequency of race Av5-6-7 (74%) greater than that of race Av5-7 (26%).

### Detection of *Rlm* genes and quantitative resistance in different cultivars in controlled-environment experiments

In cotyledon inoculation experiments (Table 2), at 18 dpi Adriana and Bilbao showed resistant phenotypes against isolates 99–79 (Av2-4-7) and v23.2.1 (Av4-5-6-7) with disease scores ranging from 1.0 to 2.3, but were susceptible to isolate v23.11.9 (Av1-5-6-7) with disease scores ranging from 7.7 to 8.6, confirming that these two cultivars carry the resistance gene *Rlm4*. Similarly, Capitol and DK Cabernet showed resistant phenotypes against isolates v23.11.9 (Av1-5-6-7) and ME24 (Av1-6-7) with disease scores ranging from 1.3 to 1.6 but were susceptible to isolate v23.2.1 (Av4-5-6-7) with disease scores ranging from 7.8 to 8.8, confirming that these two cultivars carry the resistance gene *Rlm1*; Excel and Roxet showed resistant phenotypes against all four isolates (carrying *AvrLm7*) with disease scores ranging from 1.0 to 1.6, suggesting that these two cultivars carry resistance gene *Rlm7*. DH lines A30 and C119 were susceptible to isolates 99–79 (Av2-4-7), v23.11.9 (Av1-5-6-7) and ME24 (Av1-6-7) with disease scores ranging from 7.8 to 8.8.

In petiole inoculation experiments (Table 2) to test stem canker development on Capitol and DK Cabernet or Bilbao and Adriana with A30 and C119 as controls, at 25 dpi all the inoculated leaves (i.e. those whose petioles had been inoculated) of A30 were dead or had turned yellow, while 16% of inoculated leaves of C119 were still green. Some inoculated leaves of Capitol (11%), DK Cabernet (22%), Bilbao (16%) and Adriana (22%) were still green. At 35 dpi, all the inoculated leaves had abscised and phoma stem canker symptoms were visible at the leaf scars of the inoculated leaves of the susceptible control A30, with 72% of the leaf scars showing phoma stem canker symptoms compared with 33% of the leaf scars of C119. There were some leaf scars of Capitol (16%) showing phoma stem canker symptoms but no phoma stem canker symptoms were observed on the leaf scars of DK Cabernet. There were 50% and 6% of leaf scars of Bilbao and Adriana, respectively, that showed phoma stem canker symptoms.

**Fig 7:**
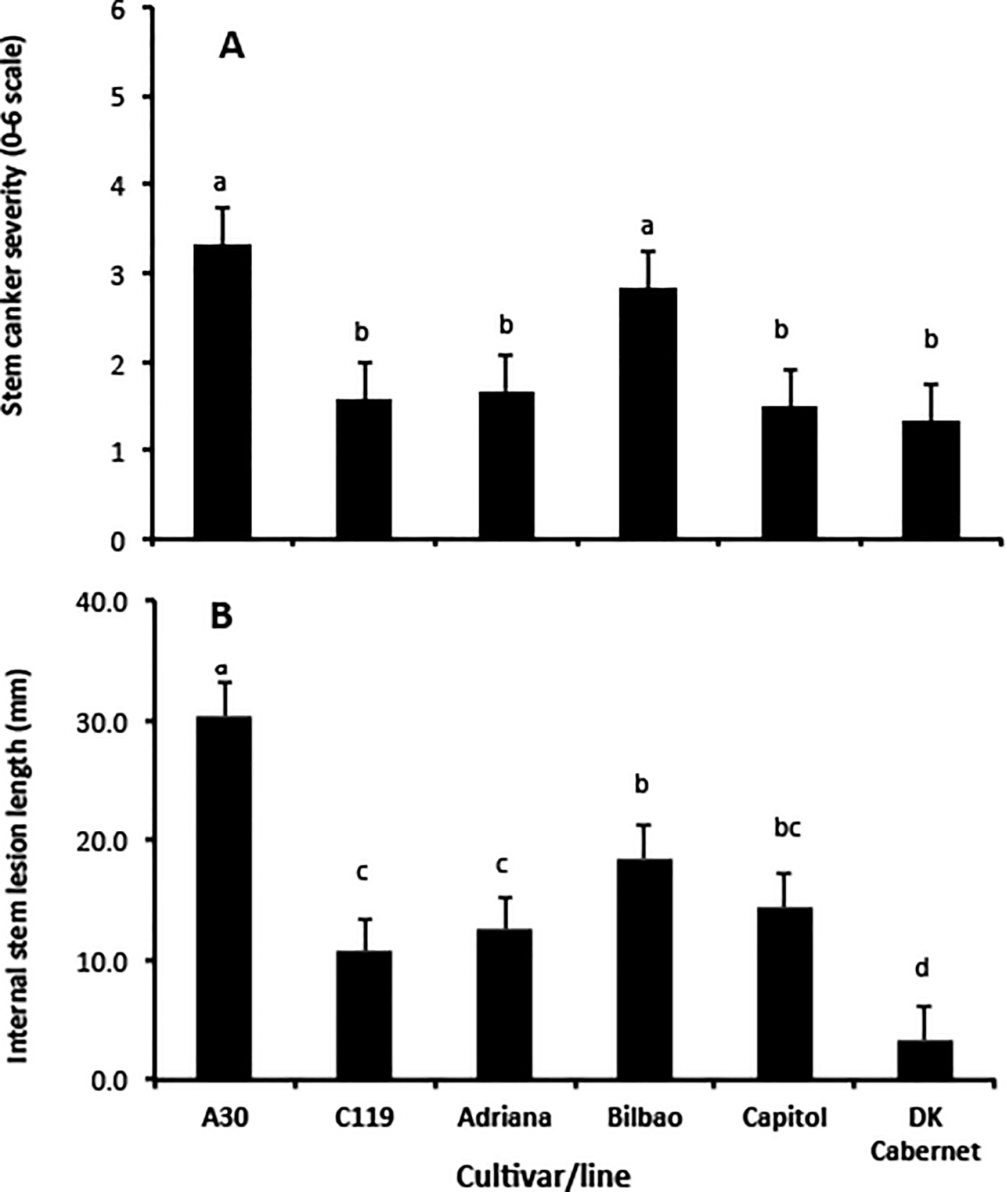
Phoma stem canker severity in controlled environment experiment. Cultivar difference in the severity of phoma stem canker scored on a 0–6 scale (A) and the internal length of phoma stem canker symptoms (B) that had developed at 20°C by 49 days after inoculation of leaf petioles of different cultivars/lines with conidial suspensions of *Leptosphaeria maculans* isolates. For details of isolates, see Table 2. Error bars are standard errors of the difference. Multiple comparisons were done using the least significance difference at significance level *P*≤0.05. The values for phoma stem canker severity or the internal length of phoma stem canker symptoms that had different letters differed significantly at *P*≤0.05.

**Fig 8:**
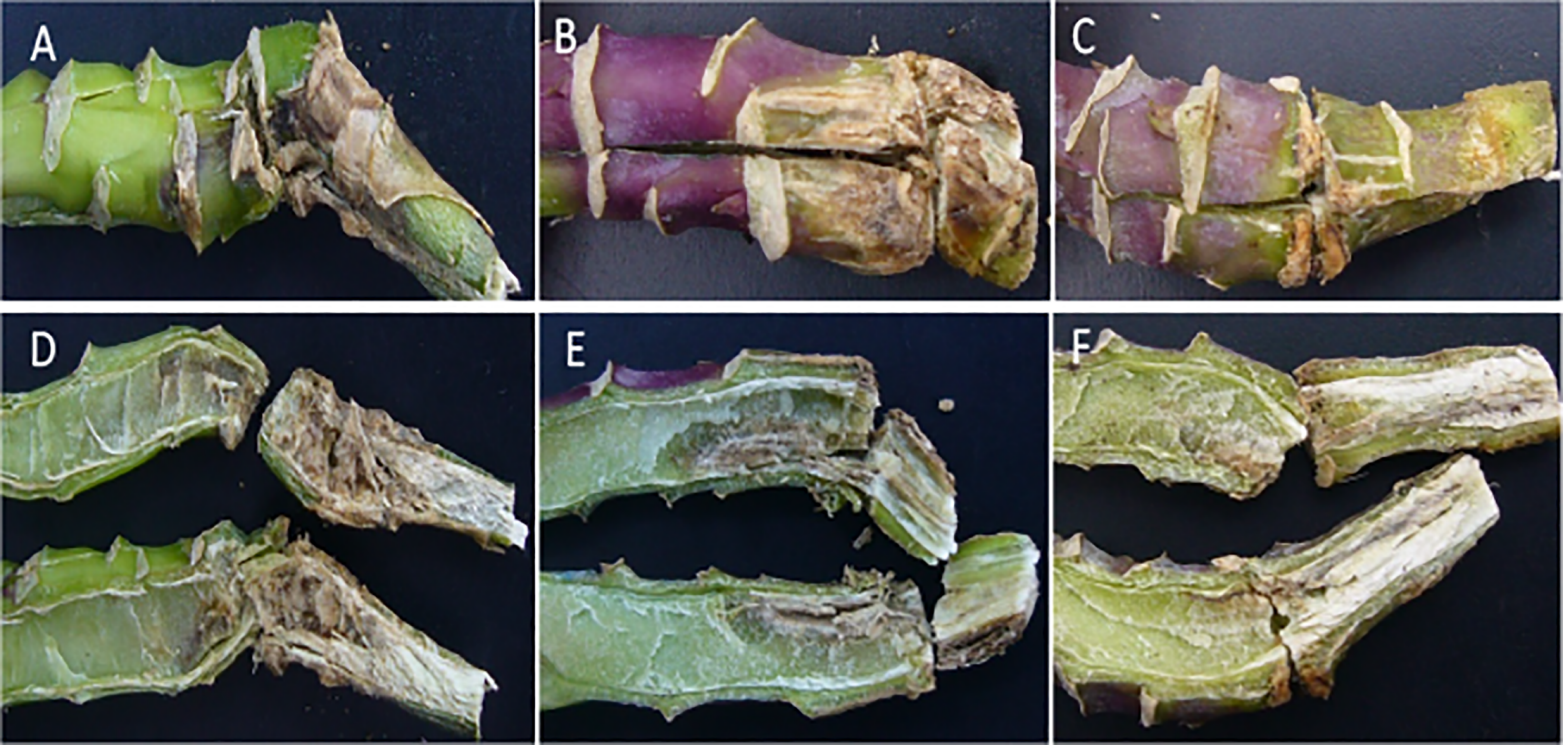
Phoma stem canker symptoms that developed in a controlled environment experiment. External (A, B, C) and internal (D, E, F) phoma stem canker symptoms on DH line A30 (A, D), cultivars Bilbao (B, E) and Adriana (C, F). A30 with no known *Rlm* resistance genes and no quantitative resistance (QR), Bilbao (*Rlm4*) with resistance gene *Rlm4*, Adriana (*Rlm4* + QR) with *Rlm4* and QR. Leaf petioles were inoculated with conidia of *Leptosphaeria maculans* isolate v23.11.9, which is virulent against *Rlm4*. Inoculated plants were kept at 20°C with 12h light/12h darkness and sampled at 49 days after inoculation.

Severity of phoma stem canker was assessed at 49 dpi; there was a significant difference (*P* < 0.01, 5 d.f., SED 0.41) between cultivars, with stem canker more severe on A30 and Bilbao than on the other four cultivars (Fig 7A). Phoma stem canker was more severe on Bilbao than on Adriana but there was no difference between Capitol and DK Cabernet. After it reached the stem, *L*. *maculans* was observed to spread vertically up/down the stem and horizontally towards the stem pith (Fig 8). Necrotic stem canker symptoms were observed in the pith of all plants of A30 and Bilbao but only in the pith of some plants of Adriana (50%), DK Cabernet (17%), Capitol (50%) and C119 (33%). There was also a significant difference (*P* < 0.01, 5 d.f., SED 2.80) between cultivars in the length of internal phoma stem canker necrosis, with A30 having the longest internal necrosis (Fig 7B). There were significant differences between Adriana and Bilbao or between DK Cabernet and Capitol in length of internal stem necrosis. Phoma stem canker necrosis had spread further along the internal stem tissues of Capitol than along those of DK Cabernet; it had spread further along the internal stem tissues of Bilbao than along those of Adriana (Fig 7B; Fig 8E and 8F).

## Discussion

This study has provided evidence that combining *R* gene resistance and QR can increase effectiveness of cultivar resistance against *L*. *maculans* in different environments. Results of field experiments at 13 different sites over three growing seasons showed that cultivars with resistance genes *Rlm1* or *Rlm4* combined with QR (e.g. DK Cabernet and Adriana, respectively) had significantly less severe phoma stem canker than cultivars with only *Rlm1* or *Rlm4* without QR (e.g. Capitol and Bilbao, respectively). This is in agreement with previous work on the resistance gene *Rlm6*, which was effective for control of phoma stem canker for up to 7 years when it was introduced into a background with QR (i.e. cultivar Darmor) [17,31]. By contrast, this resistance gene was not effective after 3 years when it was introduced into a background without QR (i.e. cultivar Eurol). However, previous work on *Rlm6* was done at only one experimental site and *Rlm6* has never been used in commercial cultivars. This study tested three different *Rlm* genes (*Rlm1*, *Rlm4* and *Rlm7*) used in commercial cultivars in 13 different environments over three growing seasons. Therefore, this work can provide more useful information for breeders and growers. Results of this study showed that resistance in cultivars with an *Rlm* gene and QR (e.g. DK Cabernet, Adriana and Excel) was less sensitive to a change in environment than that in cultivars with only *R* genes (e.g. Capitol, Bilbao) or only QR (e.g. DK Grandia), suggesting that combining *R* gene and QR can provide effective, stable resistance for control of phoma stem canker in different environments.

The presence of QR in cultivars DK Cabernet and Adriana was confirmed by petiole inoculation with *L*. *maculans* isolates virulent against *Rlm1* or *Rlm4*. In controlled conditions, Bilbao developed more severe stem canker than Adriana in terms of stem canker severity score or internal growth of *L*. *maculans* along the stem; there was no difference between Capitol and DK Cabernet in stem canker severity score but there was a difference between them in internal growth of *L*. *maculans* along the stem. This confirmed previous evidence that QR operates by impeding growth of *L*. *maculans* in leaf petioles and stems and subsequently reducing the severity of phoma stem canker by the end of the growing season [32]. This is further supported by the evidence that inoculated leaves of cultivars with QR remained green for longer than those of the susceptible control A30. For example, at 25 dpi all inoculated leaves of A30 were dead or yellow, while 22% of inoculated leaves of DK Cabernet or Adriana were still green. To assess QR in Excel and Roxet, there is a need to use isolates virulent against *Rlm7* so that the effects of *Rlm* gene resistance can be avoided. All the *L*. *maculans* isolates collected in 2010 were avirulent against *Rlm7*. Therefore, in this study, it was not possible to assess QR in Excel and Roxet.

Differences between cultivars Es-Astrid and NK Grandia with only QR suggest that the source of QR affects the effectiveness of control of phoma stem canker. Results of field experiments showed that QR in Es-Astrid was better for controlling phoma stem canker than QR in NK Grandia. Previous studies showed that combining different sources of QR in a cultivar can achieve effective disease control [33,34]. With the increase in available genome sequences and molecular markers, pyramiding of QR or combining QR with *R* genes in breeding is becoming an important strategy for durable crop protection [35]. Compared with *Rlm* gene-mediated resistance, there has been little work to investigate different sources of QR. A major source of QR (e.g. in cultivar Darmor) used in breeding European oilseed rape cultivars is derived from Jet Neuf and there are limited available sources of QR [36–38]. In an association mapping of QR for resistance against *L*. *maculans*, a collection of 128 oilseed rape cultivars/lines was analysed with 72 molecular markers; 61 markers were associated with QR; of those markers, 52% were related to the QR from Darmor [37]. There is a need to investigate good sources of QR for combining with *R* genes to achieve effective, stable disease resistance.

Temperature and rainfall are the two major environmental factors affecting severity of phoma stem canker. Analysis of the relationship between severity of stem canker and weather data among the 13 sites in the three growing seasons showed that increased severity of stem canker was associated with increased rainfall during the phoma leaf spot development stage and increased temperature during the stem canker development stage. Because epidemics of phoma stem canker are initiated from ascospores released from pseudothecia produced on stem debris and the release of ascospores occurs only after rainfall or heavy dew [39,40], increased rainfall in August and September favours ascospore maturation and increased rainfall from October to March favours ascospore release and phoma leaf spot development. Once *L*. *maculans* has reached the stem, temperature is more important than rainfall for development of stem canker symptoms since previous work showed that increased temperature led to increased severity of stem canker [32].

Seven races were identified in the UK *L*. *maculans* population in 2010 based on results from eight *Avr* loci and races Av5-6-7 and Av4-5-6-7 were the two major races. Interestingly, the race Av5-6-7 remained the major race compared with the *L*. *maculans* populations in 2000–2001 in France [25] and 2002 in the UK [41]. The number of UK *L*. *maculans* races identified was similar to that in other European countries (France, Germany, Poland and Sweden) [25, 41] but was much smaller than that in the Americas (Canada, USA and Chile) [9–10]. For example, 35 races were identified in the Americas while only eight races were identified in Europe based on characterisation of *L*. *maculans* isolates at seven *Avr* loci [9]. Recently, 150 races were identified in western Canada based on characterisation of *L*. *maculans* isolates at 12 *Avr* loci [10]. One of the reasons for the reduced number of races in Europe may be due to continuous use of *Rlm* genes leading to the fixing of virulent alleles in the *L*. *maculans* populations [9]. For example, the frequencies of *AvrLm2* and *AvrLm9* were 0% (i.e. the virulent alleles of the two *Avr* genes were fixed in the *L*. *maculans* populations) in this study and previous studies in France [25] and the UK [41], while they were 64% and 3%, respectively, in Canada [10]. Another reason may be due to the interactions between different effector genes. Recent studies showed that host recognition of *AvrLm3* and *AvrLm9* was masked by the presence of *AvrLm4-7* [42, 43].

The UK *L*. *maculans* isolates collected in 2002 was 100% avirulent against *Rlm7* [41] and it was still 100% in the 2010, suggesting that *Rlm7* is still effective for controlling phoma stem canker in the UK even though this resistance gene has been used commercially for more than 15 years [9]. However, the frequency of *AvrLm1* remained almost the same (18% in 2002, 16% in 2010), while the frequency of *AvrLm4* increased from 7% to 41%. The increased frequency of *AvrLm4* may have been due to the fitness cost associated with evolution from avirulence to virulence at the *AvrLm4* locus in *L*. *maculans* [44]. Previous work showing that fitness cost associated with pathogen evolution from avirulence to virulence to overcome host resistance can be used to predict durability of the corresponding host resistance gene [44,45]. There is a need to investigate whether there is fitness cost associated with virulence at the *AvrLm7* locu*s*. To prevent the breakdown of *Rlm7* mediated resistance, there is a need to continue to monitor *L*. *maculans* populations and to combine this resistance with QR to achieve sustainable management of phoma stem canker in oilseed rape.

## Supporting information

S1 Table Monthly mean temperature (°C) during the three growing seasons (2010/2011, 2011/2012, 2012/2013) in winter oilseed rape field experiments at 13 sites. (DOCX)

S2 Table Monthly total rainfall (mm) during the three growing seasons (2010/2011, 2011/2012, 2012/2013) in winter oilseed rape field experiments at 13 sites. (DOCX)

S3 Table Mean temperature (°C) and total rainfall (mm) during August to September (Aug-Sept), October to November (Oct-Nov), December to March (Dec-Mar) and April to June (Apr-Jun) during the three growing seasons (2010/2011, 2011/2012, 2012/2013) in winter oilseed rape field experiments at 13 sites. (DOCX)
