# Supplementary material for: Combining R gene and quantitative resistance increases effectiveness of cultivar resistance against Leptosphaeria maculans in Brassica napus in different environments
Source: PLoS One. 2018 May 23;13(5):e0197752. doi: 10.1371/journal.pone.0197752 (PMC5965857; doi:10.1371/journal.pone.0197752)
Supplement: S1 Table — (DOCX) [file pone.0197752.s001.docx]

**S1 Table.** **Monthly mean temperature (°C) during the three growing seasons (2010/2011, 2011/2012, 2012/2013) in winter oilseed rape field experiments at 13 sites.**

| Location | Cropping  year | Aug | Sept | Oct | Nov | Dec | Jan | Feb | Mar | Apr | May | Jun | Jul |
| --- | --- | --- | --- | --- | --- | --- | --- | --- | --- | --- | --- | --- | --- |
| Bainton | 2010/11 | 14.8 | 13.3 | 9.4 | 4.3 | -1.3 | 2.8 | 5.3 | 6.0 | 10.8 | 11.2 | 13.6 | 14.2 |
| Bainton | 2012/13 | 15.8 | 12.4 | 8.4 | 5.6 | 3.2 | 2.5 | 2.1 | 1.4 | 6.6 | 9.8 | 12.5 | 16.8 |
| Banbury | 2010/11 | 15.9 | 14.0 | 10.6 | 5.3 | -0.8 | 4.0 | 6.9 | 7.2 | 11.9 | 12.7 | 14.3 | 15.8 |
| Banbury | 2011/12 | 16.4 | 15.9 | 13.1 | 9.6 | 5.8 | 5.3 | 3.7 | 7.5 | 7.1 | 11.8 | 13.8 | 15.7 |
| Banbury | 2012/13 | 17.1 | 13.2 | 9.7 | 6.5 | 4.8 | 3.5 | 3.1 | 2.8 | 7.8 | 11.1 | 14.4 | 19.0 |
| Cowlinge | 2010/11 | 16.3 | 14.3 | 11.0 | 5.5 | -0.2 | 4.2 | 6.5 | 6.9 | 12.4 | 13.2 | 15.1 | 15.7 |
| Cowlinge | 2011/12 | 16.7 | 16.5 | 13.5 | 9.7 | 5.8 | 5.6 | 3.6 | 8.0 | 7.7 | 12.3 | 14.2 | 16.3 |
| Cowlinge | 2012/13 | 17.8 | 13.8 | 9.9 | 6.8 | 4.7 | 2.5 | 2.5 | 2.6 | 7.9 | 10.4 | 13.9 | 18.8 |
| Harper Adams | 2011/12 | 15.9 | 15.7 | 12.8 | 9.3 | 5.9 | 5.2 | 4.0 | 8.2 | 7.2 | 11.9 | 13.8 | 15.6 |
| Harpenden | 2010/11 | 15.8 | 13.8 | 10.5 | 5.1 | -0.3 | 3.8 | 6.2 | 6.5 | 12.2 | 12.5 | 14.3 | 15.4 |
| Harpenden | 2011/12 | 15.8 | 15.3 | 12.8 | 9.3 | 5.7 | 5.5 | 3.3 | 8.0 | 7.4 | 12.0 | 13.8 | 15.7 |
| Harpenden | 2012/13 | 17.2 | 13.3 | 9.7 | 6.5 | 4.5 | 2.7 | 2.7 | 2.5 | 7.6 | 10.5 | 13.7 | 18.7 |
| Horncastle | 2011/12 | 16.3 | 15.8 | 12.6 | 8.6 | 5.4 | 5.1 | 3.6 | 8.0 | 7.4 | 11.6 | 13.7 | 15.8 |
| Horncastle | 2012/13 | 16.9 | 13.3 | 9.2 | 6.3 | 4.0 | 2.7 | 2.8 | 2.3 | 7.6 | 10.6 | 13.8 | 18.4 |
| Morley | 2010/11 | 16.1 | 13.9 | 10.9 | 5.7 | -0.1 | 4.2 | 6.2 | 6.3 | 11.6 | 13.3 | 15.3 | 15.3 |
| Morley | 2011/12 | 16.2 | 15.8 | 12.6 | 9.2 | 5.4 | 5.3 | 3.9 | 7.6 | 7.3 | 11.9 | 13.9 | 15.9 |
| Morley | 2012/13 | 17.6 | 13.5 | 9.8 | 6.7 | 4.6 | 2.7 | 2.7 | 2.8 | 7.7 | 10.3 | 13.8 | 18.2 |
| Oadby Lodge | 2011/12 | 16.5 | 16.0 | 12.9 | 9.4 | 6.1 | 5.6 | 4.1 | 7.9 | 7.4 | 11.8 | 13.9 | 15.8 |
| Rothwell | 2010/11 | 15.8 | 14.2 | 10.4 | 5.2 | -0.7 | 3.7 | 6.2 | 6.8 | 12.0 | 12.6 | 14.6 | 15.9 |
| Rothwell | 2011/12 | 16.2 | 15.8 | 12.6 | 8.8 | 5.5 | 5.4 | 3.9 | 8.2 | 7.4 | 11.7 | 13.8 | 16.0 |
| Rothwell | 2012/13 | 17.0 | 13.4 | 9.2 | 6.4 | 4.2 | 3.0 | 2.9 | 2.3 | 7.9 | 10.9 | 13.9 | 18.5 |
| Spalding | 2010/11 | 15.9 | 14.1 | 10.7 | 5.1 | -0.5 | 3.8 | 6.4 | 6.8 | 12.2 | 12.7 | 14.7 | 15.7 |
| Spalding | 2011/12 | 16.1 | 15.7 | 13.0 | 9.1 | 5.6 | 5.3 | 3.6 | 7.9 | 7.5 | 11.9 | 13.7 | 15.7 |
| Spalding | 2012/13 | 17.2 | 13.4 | 9.5 | 6.4 | 4.5 | 2.7 | 2.9 | 2.4 | 7.6 | 10.7 | 14.0 | 18.7 |
| Stockbridge | 2010/11 | 16.0 | 14.2 | 10.9 | 5.9 | 0.2 | 4.3 | 6.6 | 7.4 | 12.7 | 12.6 | 14.4 | 15.7 |
| Stockbridge | 2012/13 | 17.2 | 13.6 | 10.3 | 6.9 | 5.5 | 4.1 | 3.2 | 3.6 | 7.8 | 10.7 | 14.6 | 19.1 |
| Bad-Salzuflen | 2010/11 | 17.3 | 13.2 | 9.7 | 4.8 | -3.6 | 2.0 | 2.5 | 5.5 | 12.4 | 14.2 | 16.5 | 16.6 |
| Bad-Salzuflen | 2011/12 | 18.1 | 16.2 | 11.0 | 6.7 | 5.0 | 3.2 | -1.0 | 8.2 | 8.7 | 14.6 | 15.2 | 17.6 |
| Bad-Salzuflen | 2012/13 | 18.1 | 14.0 | 10.2 | 6.4 | 3.1 | 1.1 | 0.6 | 1.0 | 8.6 | 12.2 | 15.9 | 18.9 |
| Verpillieres | 2010/11 | 17.5 | 14.8 | 11.2 | 6.3 | -0.2 | 4.2 | 5.8 | 7.9 | 13.4 | 14.5 | 16.3 | 16.5 |
| Verpillieres | 2011/12 | * | 17.0 | 13.0 | 9.2 | 6.8 | 5.8 | 1.6 | 9.1 | 8.6 | 14.0 | 15.8 | 17.6 |
| Verpillieres | 2012/13 | 18.6 | 14.4 | 11.6 | 7.0 | 5.7 | 2.1 | 2.1 | 3.6 | 8.9 | 10.9 | 15.6 | 19.6 |
| Mean^a^ |  | 16.6 | 14.5 | 11.0 | 7.0 | 3.3 | 3.9 | 3.8 | 5.7 | 9.1 | 12.0 | 14.4 | 16.9 |
| SD |  | 0.9 | 1.2 | 1.4 | 1.7 | 2.9 | 1.3 | 1.9 | 2.5 | 2.2 | 1.3 | 0.9 | 1.5 |
| CV (%) |  | 5.2 | 8.2 | 13.1 | 23.8 | 89.5 | 33.1 | 49.7 | 44.1 | 23.8 | 10.6 | 6.3 | 8.8 |

^a^The mean, standard deviation (SD) and coefficient of variation (CV, %) were calculated across sites/cropping years.
