# Supplementary material for: Combining R gene and quantitative resistance increases effectiveness of cultivar resistance against Leptosphaeria maculans in Brassica napus in different environments
Source: PLoS One. 2018 May 23;13(5):e0197752. doi: 10.1371/journal.pone.0197752 (PMC5965857; doi:10.1371/journal.pone.0197752)
Supplement: S2 Table — (DOCX) [file pone.0197752.s002.docx]

**S2 Table.** **Monthly total rainfall (mm) during the three growing seasons (2010/2011, 2011/2012, 2012/2013) in winter oilseed rape field experiments at 13 sites**.

| Location | Cropping  year | Aug | Sept | Oct | Nov | Dec | Jan | Feb | Mar | Apr | May | Jun | Jul |
| --- | --- | --- | --- | --- | --- | --- | --- | --- | --- | --- | --- | --- | --- |
| Bainton | 2010/11 | 62.2 | 112.4 | 68.8 | 95.6 | 41.0 | 49.6 | 59.4 | 5.2 | 2.8 | 40.2 | 57.4 | 76.0 |
| Bainton | 2012/13 | 96.8 | 82.6 | 86.6 | 125.8 | 112.2 | 54.6 | 34.0 | 47.8 | 11.4 | 80.6 | 61.8 | 22.6 |
| Banbury | 2010/11 | 128.5 | 34.9 | 58.9 | 32.8 | 14.3 | 33.4 | 49.7 | 5.1 | 2.4 | 38.2 | 36.4 | 34.5 |
| Banbury | 2011/12 | 53.3 | 25.5 | 27.0 | 24.4 | 43.4 | 32.6 | 19.6 | 13.6 | 95.8 | 51.6 | 118.8 | 100.6 |
| Banbury | 2012/13 | 69.0 | 45.2 | 76.0 | 88.4 | 85.0 | 48.3 | 36.0 | 49.3 | 28.6 | 62.6 | 29.0 | 35.8 |
| Cowlinge | 2010/11 | 112.8 | 56.8 | 54.8 | 32.2 | 21.2 | 64.8 | 31.0 | 5.0 | 2.2 | 13.0 | 52.6 | 42.2 |
| Cowlinge | 2011/12 | 36.2 | 19.0 | 25.0 | 23.8 | 55.2 | 41.6 | 12.4 | 29.8 | 107.0 | 44.2 | 100.6 | 127.0 |
| Cowlinge | 2012/13 | 61.0 | 32.0 | 101.0 | 82.8 | 96.8 | 29.6 | 24.2 | 29.2 | 29.2 | 55.4 | 18.8 | 34.2 |
| Harper Adams | 2011/12 | 19.4 | 14.6 | 36.6 | 44.2 | 68.4 | 43.2 | 17.0 | 15.6 | 135.2 | 36.8 | 88.8 | 121.2 |
| Harpenden | 2010/11 | 127.8 | 59.9 | 85.0 | 54.8 | 34.9 | 84.6 | 56.8 | 10.0 | 5.2 | 23.6 | 83.0 | 44.6 |
| Harpenden | 2011/12 | 81.2 | 38.6 | 25.3 | 36.6 | 82.4 | 58.0 | 24.7 | 34.6 | 168.7 | 52.7 | 166.4 | 128.4 |
| Harpenden | 2012/13 | 54.8 | 40.4 | 115.9 | 100.4 | 114.4 | 62.8 | 43.5 | 83.1 | 32.9 | 55.9 | 24.5 | 47.4 |
| Horncastle | 2011/12 | 38.4 | 9.4 | 39.3 | 23.6 | 58.2 | 36.2 | 5.6 | 20.6 | 151.6 | 46.0 | 139.4 | 71.2 |
| Horncastle | 2012/13 | 78.8 | 44.0 | 70.8 | 76.4 | 100.8 | 30.8 | 28.4 | 51.6 | 9.8 | 46.8 | 37.4 | 30.2 |
| Morley | 2010/11 | 94.3 | 77.6 | 72.8 | 87.6 | 24.7 | 61.0 | 39.2 | 10.6 | 4.2 | 14.4 | 64.4 | 55.0 |
| Morley | 2011/12 | 74.8 | 28.0 | 35.3 | 27.7 | 54.6 | 37.0 | 16.8 | 56.8 | 131.2 | 52.0 | 94.6 | 102.0 |
| Morley | 2012/13 | 55.2 | 44.6 | 83.4 | 71.8 | 79.7 | 47.6 | 38.4 | 75.8 | 17.6 | 52.8 | 14.2 | 6.8 |
| Oadby Lodge | 2011/12 | 40.0 | 23.6 | 42.6 | 43.6 | 60.4 | 54.2 | 13.4 | 23.0 | 116.4 | 27.0 | 134.9 | 108.2 |
| Rothwell | 2010/11 | 113.8 | 74.8 | 45.9 | 66.6 | 25.9 | 29.1 | 49.2 | 5.8 | 13.2 | 25.6 | 46.8 | 54.0 |
| Rothwell | 2011/12 | 59.2 | 21.0 | 36.6 | 20.6 | 62.4 | 45.6 | 8.9 | 25.0 | 155.4 | 35.8 | 121.8 | 69.6 |
| Rothwell | 2012/13 | 61.2 | 48.4 | 71.8 | 83.8 | 95.2 | 35.0 | 31.6 | 62.0 | 7.4 | 66.2 | 45.4 | 43.6 |
| Spalding | 2010/11 | 122.4 | 57.2 | 47.0 | 41.3 | 13.8 | 28.6 | 44.4 | 4.2 | 2.6 | 22.6 | 35.8 | 55.6 |
| Spalding | 2011/12 | 45.4 | 29.2 | 28.2 | 23.2 | 47.0 | 29.8 | 15.2 | 21.4 | 134.8 | 34.2 | 133.6 | 82.0 |
| Spalding | 2012/13 | 49.8 | 48.2 | 82.6 | 90.4 | 110.8 | 35.8 | 28.4 | 52.6 | 20.0 | 57.0 | 20.6 | 53.4 |
| Stockbridge | 2010/11 | 72.8 | 73.4 | 56.6 | 65.6 | 22.3 | 100.2 | 56.4 | 22.6 | 2.0 | 40.6 | 84.2 | 38.2 |
| Stockbridge | 2012/13 | 69.0 | 56.8 | 141.4 | 118.4 | 139.4 | 91.4 | 38.8 | 84.2 | 39.6 | 50.8 | 30.2 | 26.4 |
| Bad-Salzuflen | 2010/11 | 181.0 | 81.0 | 39.3 | 98.4 | 67.4 | 73.4 | 31.9 | 12.8 | 36.9 | 23.8 | 72.4 | 33.7 |
| Bad-Salzuflen | 2011/12 | 79.3 | 45.9 | 63.8 | 3.4 | 126.9 | 117.4 | 27.6 | 16.9 | 31.8 | 44.2 | 80.0 | 99.6 |
| Bad-Salzuflen | 2012/13 | 13.4 | 37.6 | 64.9 | 38.0 | 99.8 | 58.1 | 42.8 | 31.2 | 24.2 | 99.6 | 69.9 | 9.0 |
| Verpillieres | 2010/11 | 114.7 | 63.7 | 46.4 | 75.7 | 45.6 | 61.6 | 29.7 | 12.6 | 13.2 | 1.8 | 58.2 | 51.7 |
| Verpillieres | 2011/12 | * | 44.3 | 36.2 | 23.4 | 135.2 | 52.7 | 10.8 | 26.7 | 69.4 | 48.1 | 96.5 | 76.1 |
| Verpillieres | 2012/13 | 11.6 | 12.2 | 71.3 | 55.8 | 78.4 | 17.8 | 30.8 | 22.6 | 15.2 | 92.2 | 96.7 | 115.4 |
| Mean^a^ |  | 73.5 | 46.3 | 60.5 | 58.7 | 69.3 | 51.5 | 31.1 | 30.2 | 50.6 | 44.9 | 72.3 | 62.4 |
| SD |  | 37.9 | 23.6 | 27.4 | 32.2 | 36.4 | 22.6 | 14.6 | 23.3 | 55.5 | 21.4 | 40.1 | 34.7 |
| CV (%) |  | 51.6 | 50.9 | 45.3 | 55.0 | 52.5 | 44.0 | 46.8 | 77.0 | 109.7 | 47.6 | 55.4 | 55.7 |

^a^The mean, standard deviation (SD) and coefficient of variation (CV, %) were calculated across sites/cropping years.
