# Supplementary material for: Combining R gene and quantitative resistance increases effectiveness of cultivar resistance against Leptosphaeria maculans in Brassica napus in different environments
Source: PLoS One. 2018 May 23;13(5):e0197752. doi: 10.1371/journal.pone.0197752 (PMC5965857; doi:10.1371/journal.pone.0197752)
Supplement: S3 Table — (DOCX) [file pone.0197752.s003.docx]

**S3 Table.** **Mean temperature (°C) and total rainfall (mm) during August to September (Aug-Sept), October to November (Oct-Nov), December to March (Dec-Mar) and April to June (Apr-Jun) during the three growing seasons (2010/2011, 2011/2012, 2012/2013) in winter oilseed rape field experiments at 13 sites.**

| Location | Cropping  year | Temperature | | | | Rainfall | | |  |
| --- | --- | --- | --- | --- | --- | --- | --- | --- | --- |
|  |  | Aug-Sept | Oct-Nov | Dec-Mar | Apr-Jun | Aug-Sept | Oct-Nov | Dec-Mar | Apr-Jun |
| Bainton | 2010/11 | 14.0 | 6.9 | 3.2 | 11.9 | 174.6 | 164.4 | 155.2 | 100.4 |
| Bainton | 2012/13 | 14.2 | 7.0 | 2.3 | 9.6 | 179.4 | 212.4 | 248.6 | 153.8 |
| Banbury | 2010/11 | 15.0 | 8.0 | 4.2 | 13.0 | 163.4 | 91.7 | 102.5 | 77.0 |
| Banbury | 2011/12 | 16.2 | 11.4 | 5.6 | 10.9 | 78.8 | 51.4 | 109.2 | 266.2 |
| Banbury | 2012/13 | 15.2 | 8.1 | 3.6 | 11.1 | 114.2 | 164.4 | 218.6 | 120.2 |
| Cowlinge | 2010/11 | 15.3 | 8.3 | 4.3 | 13.6 | 169.6 | 87.0 | 122.0 | 67.8 |
| Cowlinge | 2011/12 | 16.6 | 11.6 | 5.8 | 11.4 | 55.2 | 48.8 | 139.0 | 251.8 |
| Cowlinge | 2012/13 | 15.8 | 8.4 | 3.1 | 10.7 | 93.0 | 183.8 | 179.8 | 103.4 |
| Harper Adams | 2011/12 | 15.8 | 11.1 | 5.8 | 11.0 | 34.0 | 80.8 | 144.2 | 260.8 |
| Harpenden | 2010/11 | 14.8 | 7.8 | 4.0 | 13.0 | 187.7 | 139.8 | 186.3 | 111.8 |
| Harpenden | 2011/12 | 15.6 | 11.1 | 5.6 | 11.1 | 119.8 | 61.9 | 199.7 | 387.8 |
| Harpenden | 2012/13 | 15.3 | 8.1 | 3.1 | 10.6 | 95.2 | 216.3 | 303.8 | 113.3 |
| Horncastle | 2011/12 | 16.0 | 10.6 | 5.6 | 10.9 | 47.8 | 62.9 | 120.6 | 337.0 |
| Horncastle | 2012/13 | 15.1 | 7.8 | 3.0 | 10.7 | 122.8 | 147.2 | 211.6 | 94.0 |
| Morley | 2010/11 | 15.0 | 8.3 | 4.1 | 13.4 | 171.9 | 160.4 | 135.5 | 83.0 |
| Morley | 2011/12 | 16.0 | 11.0 | 5.5 | 11.0 | 102.8 | 63.0 | 165.2 | 277.8 |
| Morley | 2012/13 | 15.6 | 8.3 | 3.2 | 10.6 | 99.8 | 155.2 | 241.5 | 84.6 |
| Oldby Lodge | 2011/12 | 16.3 | 11.2 | 5.9 | 11.0 | 63.6 | 86.2 | 151.0 | 278.3 |
| Rothwell | 2010/11 | 15.0 | 7.9 | 3.9 | 13.1 | 188.6 | 112.5 | 110.0 | 85.6 |
| Rothwell | 2011/12 | 16.0 | 10.7 | 5.8 | 10.9 | 80.2 | 57.2 | 141.9 | 313.0 |
| Rothwell | 2012/13 | 15.2 | 7.8 | 3.1 | 10.9 | 109.6 | 155.6 | 223.8 | 119.0 |
| Spalding | 2010/11 | 15.0 | 8.0 | 4.1 | 13.2 | 179.6 | 88.3 | 91.0 | 61.0 |
| Spalding | 2011/12 | 15.9 | 11.1 | 5.6 | 11.0 | 74.6 | 51.4 | 113.4 | 302.6 |
| Spalding | 2012/13 | 15.3 | 8.0 | 3.1 | 10.8 | 98.0 | 173.0 | 227.6 | 97.6 |
| Stockbridge | 2010/11 | 15.1 | 8.4 | 4.6 | 13.2 | 146.2 | 122.2 | 201.5 | 126.8 |
| Stockbridge | 2012/13 | 15.4 | 8.6 | 4.1 | 11.0 | 125.8 | 259.8 | 353.8 | 120.6 |
| Bad-Salzuflen | 2010/11 | 15.3 | 7.3 | 1.6 | 14.4 | 262.0 | 137.7 | 185.5 | 133.1 |
| Bad-Salzuflen | 2011/12 | 17.0 | 8.9 | 3.9 | 12.8 | 125.2 | 67.2 | 287.7 | 157.1 |
| Bad-Salzuflen | 2012/13 | 15.2 | 8.3 | 1.5 | 12.2 | 51.0 | 102.9 | 231.9 | 193.7 |
| Verpillieres | 2010/11 | 16.2 | 8.8 | 4.4 | 14.7 | 178.4 | 122.1 | 149.5 | 73.2 |
| Verpillieres | 2011/12 | 17.0 | 11.1 | 5.9 | 12.8 | 44.3 | 59.6 | 225.4 | 214.0 |
| Verpillieres | 2012/13 | 16.5 | 9.4 | 3.4 | 11.8 | 23.8 | 127.1 | 149.6 | 204.1 |
| Mean^a^ |  | 15.6 | 9.0 | 4.1 | 11.8 | 117.5 | 119.2 | 182.1 | 167.8 |
| SD |  | 0.7 | 1.5 | 1.3 | 1.3 | 56.3 | 55.2 | 63.0 | 92.4 |
| CV (%) |  | 4.5 | 16.5 | 30.8 | 10.8 | 47.9 | 46.3 | 34.6 | 55.1 |

^a^The mean, standard deviation (SD) and coefficient of variation (CV, %) were calculated across sites/cropping years.
